# MicroRNA-3619-5p suppresses bladder carcinoma progression by directly targeting β-catenin and CDK2 and activating p21

**DOI:** 10.1038/s41419-018-0986-y

**Published:** 2018-09-20

**Authors:** Qingsong Zhang, Shuo Miao, Xihong Han, Chuanchang Li, Mengyang Zhang, Kai Cui, Tao Xiong, Zhong Chen, Chenghe Wang, Hua Xu

**Affiliations:** 1 Department of Urology, Tongji Hospital, Tongji Medical College, Huazhong University of Science and Technology, No. 1095 JieFang Avenue, 430030 Wuhan, Hubei China; 2 Department of Urology, The Affiliated Hospital of Qingdao University, No. 16 Jiangsu Road, Shinan District, 26600 Qingdao, Shandong China; 3 Department of Pharmacology, Tongji Medical College, Huazhong University of Science and Technology, No. 1095 JieFang Avenue, 430030 Wuhan, Hubei China; 4 Department of Cardiology, Shouguang People’s Hospital, 262700 Shouguang, Shandong China; 5 Department of Urology, Ruijin Hospital, School of Medicine, Shanghai Jiaotong University, 200025 Shanghai, China

## Abstract

Current studies indicate that microRNAs (miRNAs) are widely decreased in various tumors and function as tumor suppressors by inhibiting cancer cell proliferation, survival, invasion, and migration. The potential application of using miRNAs to predict therapeutic responses to multiple types of cancer treatment holds high promise. In current study, we demonstrate that miR-3619-5p is downregulated in bladder cancer (BCa) tissues and cells. Exogenous overexpression of miR-3619-5p in BCa cells inhibits proliferation, migration, and invasion. Moreover, a nude mouse xenograft model shows that miR-3619-5p inhibits BCa cell growth. We also demonstrate that miR-3619-5p leads to the activation of p21 by targeting its promoter in BCa cells. Enforced miR-3619-5p expression consistently leads to the downregulation of β-catenin and cyclin-dependent kinase 2 (CDK2) through predicted binding sites within the β-catenin and CDK2 3′-untranslated regions (UTRs), respectively. Moreover, β-catenin and CDK2 knockdown is able to mimic BCa cells growth and metastasis effects induced by overexpressing miR-3619-5p. We further confirm that miR-3619-5p inhibits Wnt-β-catenin signal pathway and EMT progression in BCa cells. We also found that miR-3619-5p-induced growth arrest and metastasis inhibition are p21-dependent in BCa cells. Taken together, these results confirm that miR-3619-5p plays a tumor suppressive role in BCa by interfering with cell growth and metastasis and may serve as a potential therapeutic target in BCa treatment.

## Introduction

Bladder cancer (BCa) is one of the most common urological malignancy, and the incidence of BCa is expected to rise globally^1^. There are approximate 430,000 newly diagnosed cases every year all over the world and BCa is a common cause of cancer-related death among urinary tumors in China^2^. Although multiple treatments have been gained, the 5-year survival rate of BCa patients is still dissatisfied^3^. About 33−75% of patients with BCa failed to respond to therapy due to the disease relapse or metastasis^4^. There is an urgent need for further investigation of the carcinogenesis and development of BCa. Regulation of specific tumor suppressor genes was confirmed to largely contribute to BCa initiation, proliferation, and metastasis; these results have led the scholars to research novel therapies based on targeted gene therapy for cancer treatment^5^.

miRNAs are a cluster of small endogenous noncoding RNAs composed of approximately 19−24 nucleotides that regulate target genes post-transcriptionally^6^. miRNAs play a key role in tumor cells growth, differentiation, metastasis, and apoptosis^7,8^. Increasing evidence has shown that miRNAs are involved in the progression of multiple types of cancers, including hepatocellular carcinoma, gastric cancer, glioma, and BCa^9–12^. In this regard, miRNAs are considered to be pivotal regulators of genes expression.

It is recently reported that the Wnt/β-catenin signaling pathway is associated with BCa cell proliferation and differentiation^13^. Additionally, miRNAs are able to inhibit BCa cell epithelial−mesenchymal transition (EMT), which plays a crucial role in the early stages of proliferation and invasiveness^14,15^. In this study, we discovered that miR-3619 was decreased in both BCa cell lines and BCa clinical specimens. Enforced miR-3619 expression interfered with cell proliferation and metastasis and promoted cellular senescence and apoptosis; tumor growth in vivo was also suppressed. Furthermore, BCa cell proliferation and metastasis abilities were boosted by silencing endogenous miR-3619. Moreover, we demonstrated that CDK2 and β-catenin, both of which are direct miR-3619 target genes, played very important roles in BCa cell growth and metastasis. We also confirmed that miR-3619 activated p21 expression, which has a potent ability to suppress BCa progression^16^ by binding to its specific promoter. Together, our results provided new evidence that miR-3619 overexpression inhibited BCa progression and might represent a novel therapeutic target for BCa treatment.

## Results

### miR-3619 and p21 expression are reduced in both BCa tissues and BCa cell lines and associated with cancer progression

**Fig. 1:**
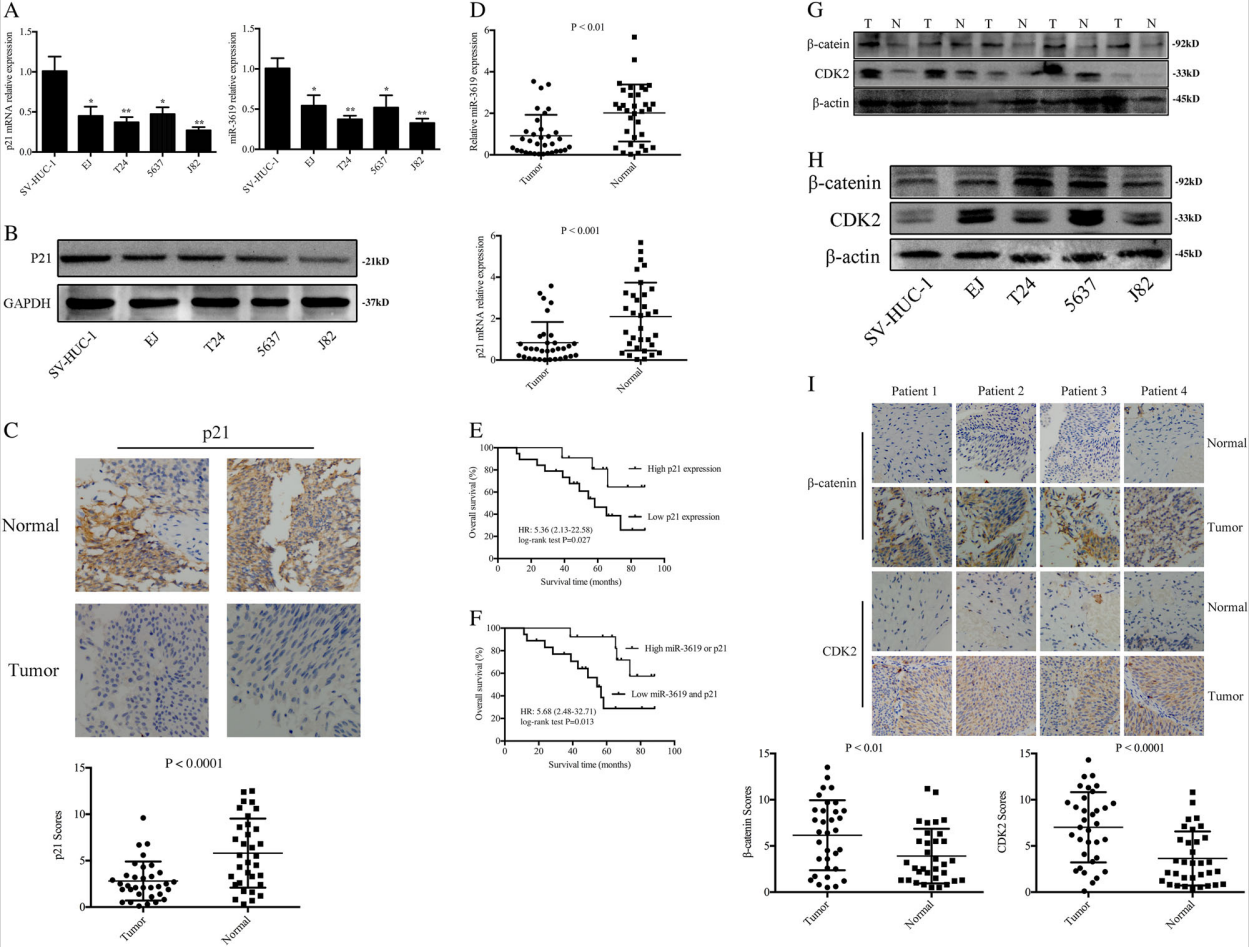
The expression of miR-3619 and p21 as well as β-catenin and CDK2 are detected in BCa cells and tissues. **a** P21 mRNA and miR-3619 expression were reduced in 5637, T24, EJ, and J82 cell lines compared with primary normal human bladder epithelial cells (SV-HUC-1) (*P < 0.05 and **P < 0.01). **b** P21 protein expression in BCa cells was assessed by western blot analysis. GAPDH served as a loading control. **c** P21 protein expression in BCa tissues was detected by IHC assay (400×). **d** P21 mRNA and miR-3619 expression were reduced in human BCa tissues compared with their expression in adjacent normal tissues. **e** Kaplan−Meier curves and the log-rank test showed that high p21 expression was associated with a favorable OS compared with low p21 expression (*P* = 0.027). **f** Kaplan−Meier curves and the log-rank test indicated that patients with low miR-3619 and p21 expression levels had significantly shorter survival than those with high miR-3619 or p21 expression levels (*P* = 0.013). **g**, **h** β-catenin and CDK2 protein levels were upregulated in BCa cells and tissues compared with normal bladder mucosal epithelial cells and para-cancerous tissue respectively. **i** β-catenin and CDK2 protein expression in BCa tissues was detected by IHC assay (400×)

As shown in Fig. 1a, b, miR-3619 and p21 mRNA and protein levels were significantly downregulated in four BCa cell lines (5637, EJ, T24, and J82) compared with bladder mucosa epithelial cell line SV-HUC-1 cells. In BCa tissues, the mean score of p21 in tumor tissues was much lower than that in normal tissues, 2.806 ± 0.3649 vs. 5.812 ± 0.6483 (*P* < 0.0001, Wilcoxon signed rank test) (Fig. 1c). And the average expression levels of miR-3619 and p21 mRNA levels were also lower in the BCa tissues than in the adjacent normal tissues (Fig. 1d). Spearman’s correlation test was used to evaluate the relationship between miR-3619 and p21 expression levels. The outcomes showed that miR-3619 expression was positively correlated with p21 expression (Supplementary Figure 1A, Tumor, *r* = 0.64, *P* < 0.0001; Normal, *r* = 0.61, *P* = 0.0016). Besides, miR-3619 and p21 expression (T/N) were correlated with tumor stage and grade (*P* < 0.05) and BCa clinicopathological factors were discussed and shown in Table 1. These data indicated that miR-3619 and p21 might act as tumor suppressors in BCa.

**Table 1:** Associations between expression of miR-3619, p21 and clinicopathologic factors in patients with BCa (*n* = 33)

Parameter	Number of case	miR-3619 expression	*P* value	p21expression	*P* value
Age, y					
>60	19	0.51 ± 0.31	0.83	0.58 ± 0.24	0.54
≤60	14	0.49 ± 0.26		0.66 ± 0.29	
Sex					
Male	20	0.42 ± 0.24	0.47	0.68 ± 0.22	0.96
Female	13	0.47 ± 0.27		0.70 ± 0.17	
Tumor diameter, cm					
≤3	19	0.52 ± 0.27	0.13	0.69 ± 0.25	0.17
>3	14	0.43 ± 0.22		0.56 ± 0.33	
Stage					
Ta	15	0.57 ± 0.22	0.024	0.72 ± 0.25	0.006
T1	13	0.44 ± 0.17		0.55 ± 0.15	
T2-4	5	0.37 ± 0.11		0.43 ± 0.18	
Grade					
G1	23	0.54 ± 0.28	0.022	0.75 ± 0.21	0.009
G2/G3	10	0.35 ± 0.16		0.47 ± 0.28	

### Low expression of p21 gene or decrease of both p21 and miR-3619 is associated with poor overall survival (OS) in BCa patients

We analyzed the relationship between p21 gene expression and BCa patients’ OS. The median fold change (T/N) in p21 expression used as cutoff value; patients with high p21 expression had longer survival time than patients with low p21 expression (Fig. 1e, HR = 5.36, 95% CI 2.13−22.58; *P* = 0.027)^17^. Multivariate Cox analysis was performed to detect whether p21 gene was an independent prognostic factor in BCa patients. The outcomes showed that downregulated p21 was associated with poor OS in BCa (HR = 4.38, 95% CI 1.36−11.02; *P* = 0.048) independent of other covariates (Table 2), which suggested that p21 gene in BCa patients might be an independent prognostic factor. Furthermore, the Kaplan−Meier curves demonstrated that high expression of p21 or miR-3619 in BCa patients survived longer than patients with low p21 and miR-3619 expression (Fig. 1f, HR = 5.68, 95% CI 2.48−32.71; *P* = 0.013). Similarly, the multivariate Cox regression model displayed that there was a positive correlation between the low levels of miR-3619 and p21 expression and the poor OS in BCa patients (HR = 5.66, 95% CI 1.05−23.64; *P* = 0.036) independent of other clinicopathological factors (Table 2). Additionally, the univariate and multivariate Cox regression analysis results showed that tumor grades (G2−3) and TNM stage (T2−4) were associated with poor OS (*P* < 0.05) in the 33 BCa patients.

**Table 2:** Univariate and multivariate analysis of various prognostic variables and overall survival (OS) in patients with BCa (*n* = 33)

Variables (and stratification)	Univariate analysis		Multivariate analysis	
	HR (95% CI)	*P* value	HR (95% CI)	*P* value
Age (>60 vs. ≤60 y)	1.42 (0.64−2.38)	0.55	—	—
Sex (male vs. female)	1.11 (0.55−3.61)	0.68	—	—
BMI (>25.51 vs. <25.51 kg/m^2^)	2.57 (0.77−5.80)	0.17	—	—
Stage (T2-4 vs. Ta-1)	5.74 (1.35−18.72)	0.014	5.15 (1.33−15.24)	0.023
Grade (G2-3 vs. G1)	6.31 (2.21−24.58)	0.0087	5.72 (1.28−19.83)	0.019
Tumor size (>3 vs. ≤3 cm)	4.14 (1.27−15.90)	0.074	—	—
miR-3619 expression (low vs. high)	4.27 (1.09−12.75)	0.053	—	—
p21 expression (low vs. high)	5.36 (2.13−22.58)	0.027	4.38 (1.36−11.02)	0.048
Low miR-3619 and p21 vs. high miR-3619 or p21	5.68 (2.48−32.71)	0.013	5.66 (1.05−23.64)	0.036

### β-catenin and CDK2 are overexpressed in human BCa tissues and cell lines and negatively correlated with miR-3619 expression

The expression of β-catenin and CDK2 proteins was remarkably increased in the four BCa cell lines compared with SV-HUC-1 cells (Fig. 1g). IHC and western blotting were performed to detect the protein expression characteristics of β-catenin and CDK2 in the BCa and para-carcinoma tissues. The western blotting results showed that β-catenin and CDK2 proteins had higher expression in the BCa tissues than in the adjacent normal tissues (Fig. 1h). The IHC results showed a significant enhancement of β-catenin and CDK2 expression in BCa tissues compared with adjacent normal tissues. The mean scores of β-catenin were elevated from 3.903 ± 0.5147 to 6.155 ± 0.6587 (*P* < 0.01, Wilcoxon signed rank test). Similarly, the mean scores of CDK2 in tumor tissues and adjacent normal tissues were 7.018 ± 0.6516 and 3.644 ± 0.5090, respectively (*P* < 0.0001, Wilcoxon signed rank test) (Fig. 1i). We also observed that β-catenin and CDK2 expression were significantly overexpressed in the BCa samples in comparison with the normal bladder samples using Oncomine (www.oncomine.org) from original published data (Supplementary Figure 1B). We found a strong inverse correlation between miR-3619 and β-catenin and between miR-3619 and CDK2 (Supplementary Figure 1C, *r* = −0.45, *P* = 0.009; *r* = −0.47, *P* = 0.0058), indicating the presence of a regulatory relationship between miR-3619 and CDK2/β-catenin in BCa cells.

### miR-3619 inhibits BCa cell proliferation and survival in vitro and in vivo

**Fig. 2:**
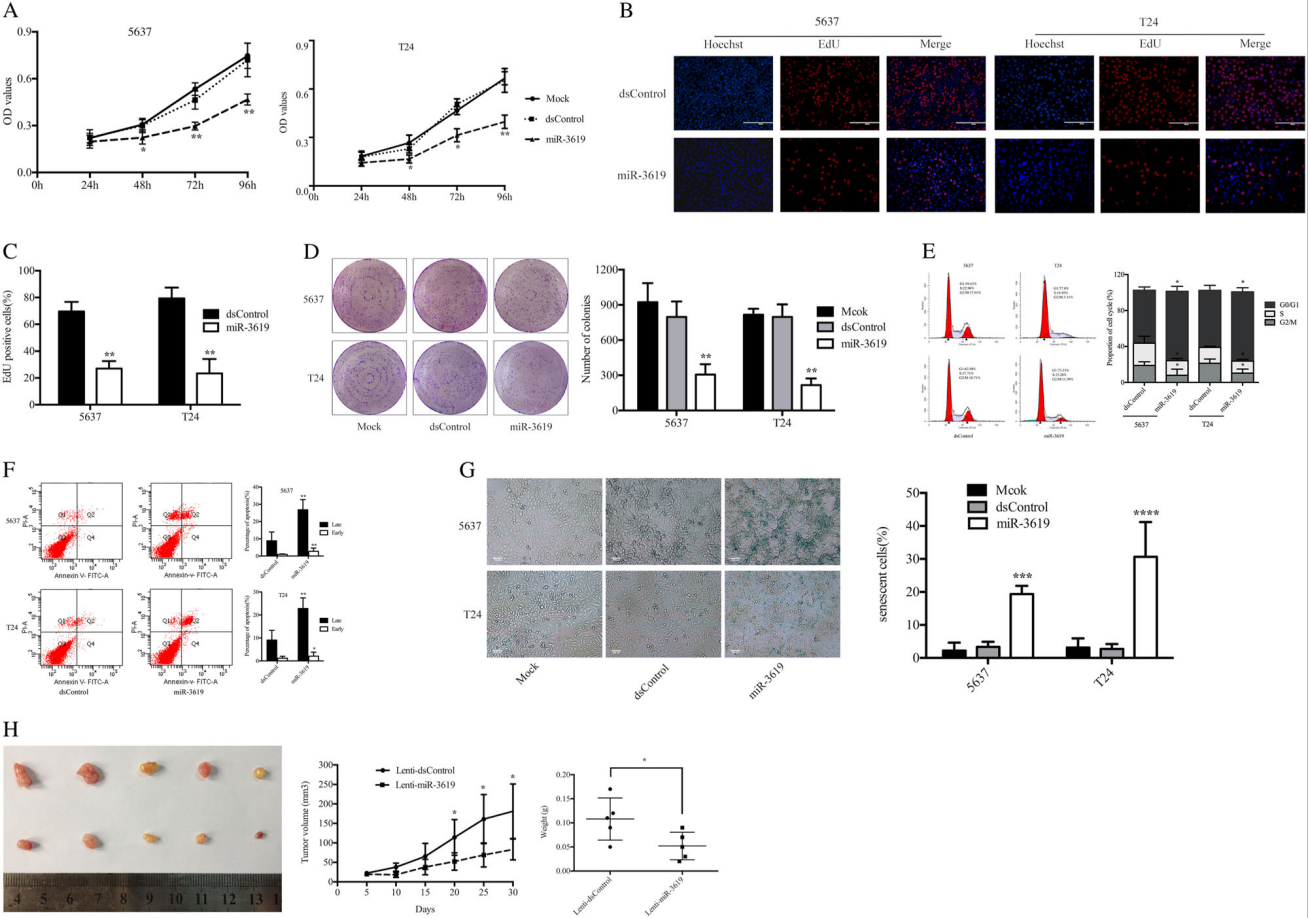
MiR-3619 inhibits BCa cell proliferation and survival in vitro and in vivo. **a** Viable cells were measured from days 1 to 4 following transfection using the CellTiter 96® AQueous One Solution Cell Proliferation Assay kit. The results are plotted as OD values. **b** Representative micrographs of EdU-positive cells (red). The nucleus was stained with DAPI (blue). **c** Quantification of EdU-positive cells. Overexpression of miR-3619 inhibited cell proliferation in both T24 and 5637 cells. **d** Representative photographs of the colony formation assay and quantification of cell colony formation. **e** Representative images of cell cycle analysis in 5637 and T24 cells as well as quantification of cell cycle distribution. **f** Representative flow cytometry images of cell apoptosis. Percentage of early and late apoptotic cells was shown by a histogram. **g** β-galactosidase assay was performed 3 days after transfection and the positively stained cells were counted. **h** Photographs of tumors excised 30 days after inoculation of stably transfected T24 cells into nude mice. The mean tumor volume measured by calipers on the indicated days. The tumor weight from each nude mouse at the end of the 28 days. *P < 0.05, **P < 0.01, ***P < 0.001, and ****P < 0.0001

As shown in Fig. 2a, compared to the dsControl group, both tested cells exhibited progressive retarded growth after 48 h as measured by CellTiter 96® AQ_ueous_ One Solution Cell Proliferation Assay. An EdU assay was conducted to further detect the alterations in cell proliferation, and the outcomes were consistent with the above results (Fig. 2b, c). The 5637 and T24 cells both showed less colony formation after enforced miR-3619 expression (Fig. 2d). Besides, the 5637 and T24 cells accumulated in G1/G0 phase and displayed reduced S phase and G2/M phase cells after miR-3619 transfection (Fig. 2e). We found that upregulation of miR-3619 increased 5637 and T24 cells’ apoptosis in both early and late stage as measured by flow cytometry with PI and Annexin V staining (Fig. 2f). Subsequently, we tested whether proliferation inhibition by miR-3619 was associated with senescence. We measured senescence-associated β-galactosidase activity (SA-β-Gal) activity and found a marked increase in positive β-galactosidase cells following miR-3619 transfection (Fig. 2g). As shown in Fig. 2h, miR-3619 overexpression remarkably delayed T24 xenograft tumor growth. Besides, the reduction and loss in the average tumor volume and weight of miR-3619-overexpressing xenografts were compared with those of the control group.

### miR-3619 restrain BCa cell migration and invasion

**Fig. 3:**
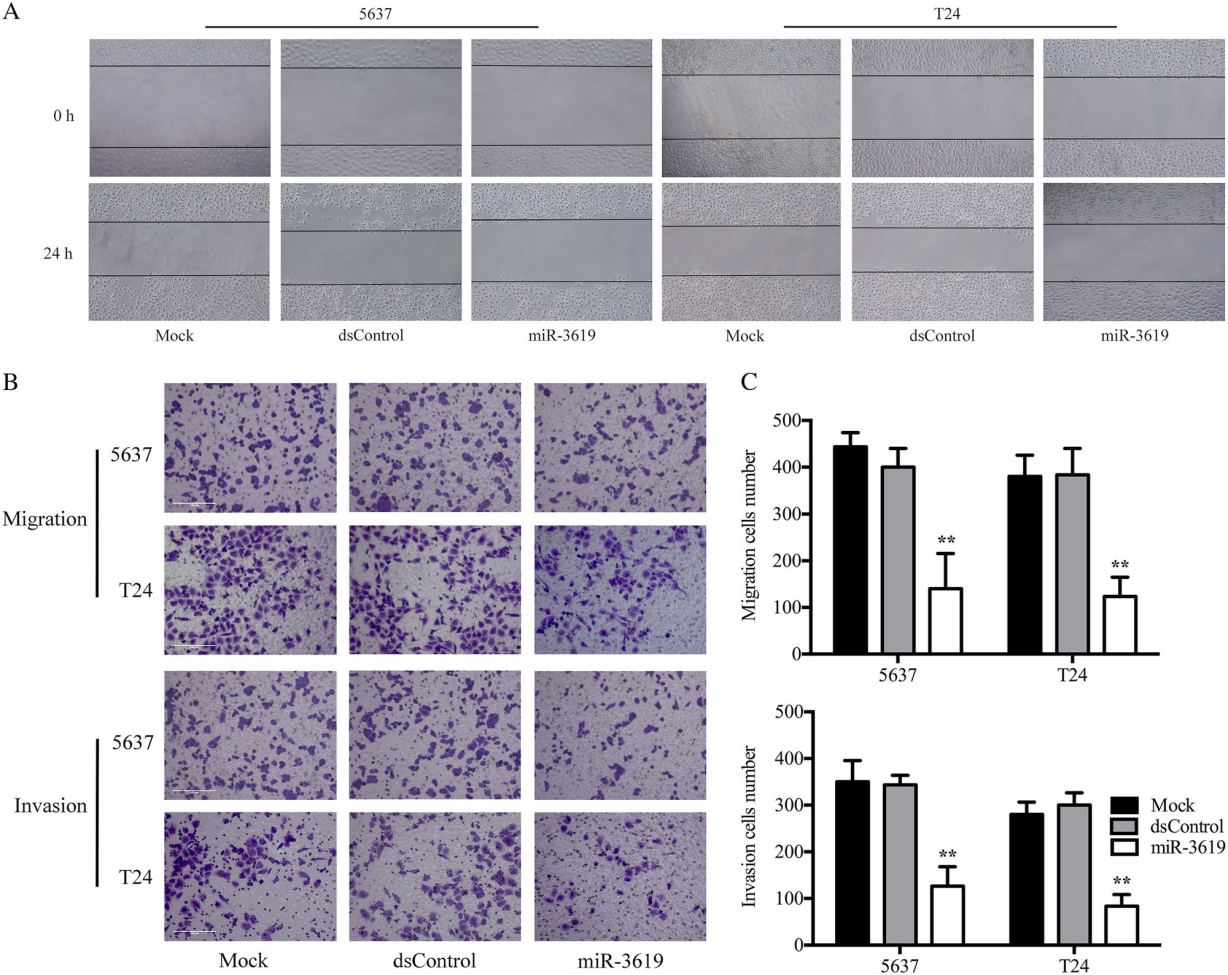
MiR-3619 suppresses BCa cell migration and invasion. T24 and 5637 cells were transfected with 50 nM miR-3619 or dsControl for 72 h. **a** Representative wound-healing images at 0 and 24 h. **b** Representative photographs of the transwell assay (200×). **c** Representative the number of migrated and invaded cells was quantified. The number of migrated and invaded cells was quantified. MiR-3619 exerted a potent inhibitory effect on migration and invasion in both cell lines. **P < 0.01 compared with the dsControl group

A wound-healing assay was performed to assess the migration capacity of 5637 and T24 cells transfected with miR-3619 mimics. The results showed that miR-3619 upregulation inhibited cell migration (Fig. 3a). We also conducted migration and Matrigel invasion chamber assays to evaluate the migration and invasion abilities of the transfected cells, respectively. In accordance with the wound-healing assay results, BCa cells transfected with miR-3619 mimics showed suppressed migration and invasion compared with the mock or dsControl group (Fig. 3b, c).

### Enforcing miR-3619 expression overexpresses p21 and downregulates β-catenin and CDK2 while inhibiting epithelial−mesenchymal transition

**Fig. 4:**
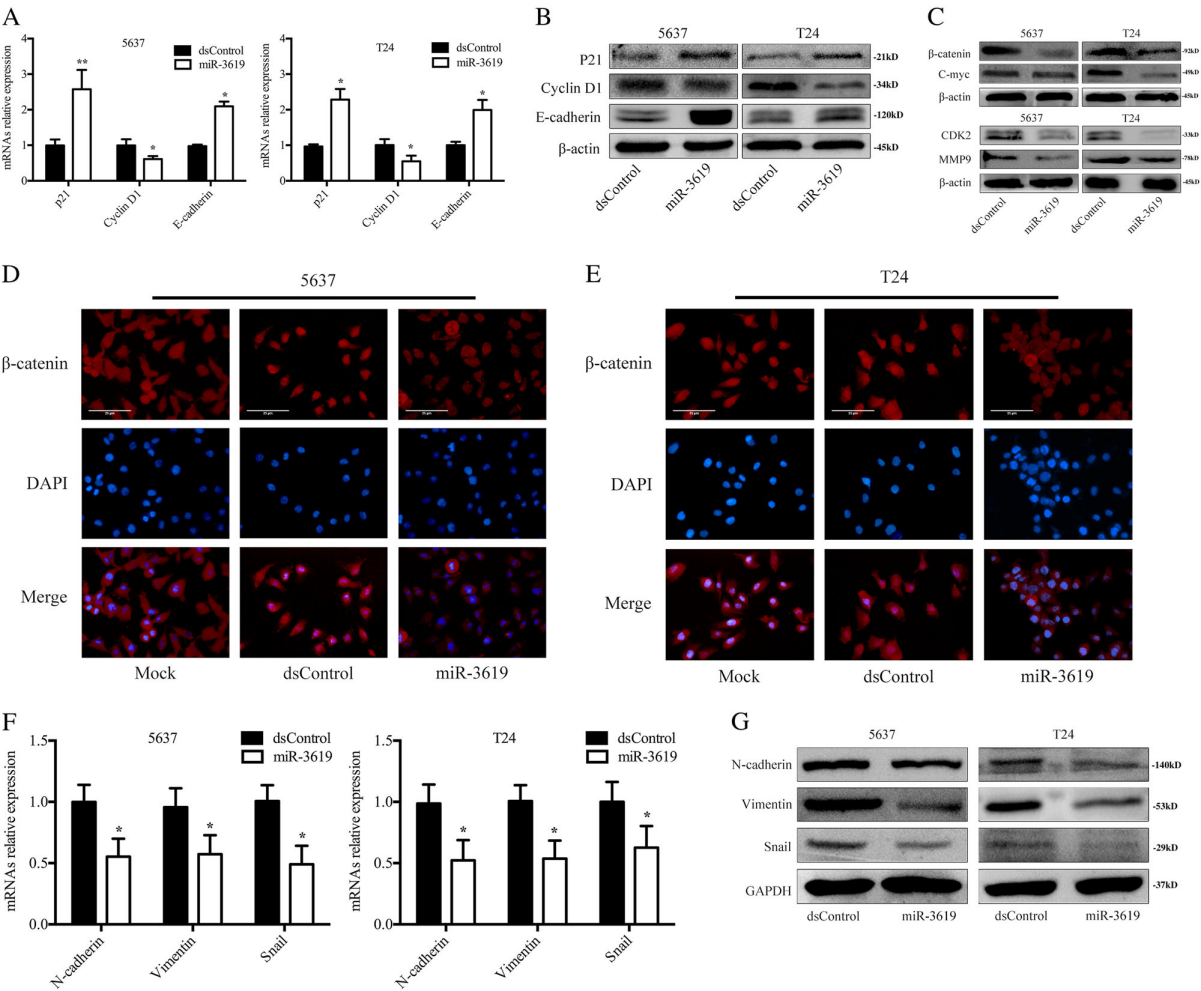
MiR-3619 induces p21, downregulates β-catenin and CDK2 expression and inhibits the EMT process. **a** p21, E-cadherin, and Cyclin D1 mRNA expression were detected by qRT-PCR. **b**, **c** The expression of proteins from potential miR-3619 targets is shown in a western blot of BCa cells from 3 days after transfection of miR-3619 or controls. **d**, **e** Immunofluorescence staining of β-catenin in 5637 and T24 cells. **f** N-cadherin, Vimentin, and Snail mRNA expression in 5637 and T24 cells were measured using qRT-PCR. **g** The N-cadherin, Vimentin, and Snail protein expression in 5637 and T24 cells were measured using western blot. *P < 0.05, **P < 0.01 compared with the dsControl group

By querying the http://www.microrna.org^18^ and TargetScan^6^ (http://www.targetscan.org) miRNA databases and searching for miRNA target screening using the miRanda algorithm^19^, we identified p21, β-catenin, and CDK2 as candidate miR-3619 targets. To confirm the influence of miR-3619 on p21, β-catenin and CDK2 in BCa cells, we transfected miR-3619 mimics into 5637 and T24 cells and analyzed p21, β-catenin and CDK2 expression after 72 h. The transfection ratios were tested by qRT-PCR (Supplementary Figure 2A). The levels of p21, β-catenin, CDK2 and each downstream target were detected after reinforced miR-3619 expression. As shown in Fig. 4a, the mRNA levels revealed a profound p21 and E-cadherin induction and Cyclin D1 suppression after miR-3619 transfection. General PCR was performed and the results were similar to the qRT-PCR results (Supplementary Figure 2B). p21, E-cadherin, and Cyclin D1 protein expression was further confirmed by immunoblot analysis (Fig. 4b). β-catenin and CDK2 protein expression were decreased and the expression of the downstream genes C-myc and MMP9 were also downregulated after miR-3619 transfection into the two cell lines (Fig. 4c).

Moreover, as β-catenin’s nuclear translocation is a hallmark that activates Wnt/β-catenin signaling^20^, immunofluorescence on 5637 and T24 cells was performed to further corroborate the nuclear localization of β-catenin. The results showed that nuclear β-catenin was decreased after miR-3619 overexpression (Fig. 4d, e). These data supported that p21, β-catenin, and CDK2 might be direct targets of miR-3619. As seen from Fig. 4a, f compared to the dsControl group, miR-3619 statistically increased the mRNA expression of the epithelial marker E-cadherin and also decreased the mesenchymal markers N-cadherin, Vimentin, and Snail in 5637 and T24 cells. General PCR was conducted and the outcomes were consistent with the qRT-PCR results (Supplementary Figure 2C). Western blotting also showed a robust upregulation in E-cadherin and downregulation in N-cadherin, Vimentin, and Snail protein levels in both 5637 and T24 cells after miR-3619 transfection (Fig. 4b, g).

### MiR-3619 interacts with the p21 gene promoter and directly targets the predicted binding sites in the β-catenin and CDK2 3′-UTRs

**Fig. 5:**
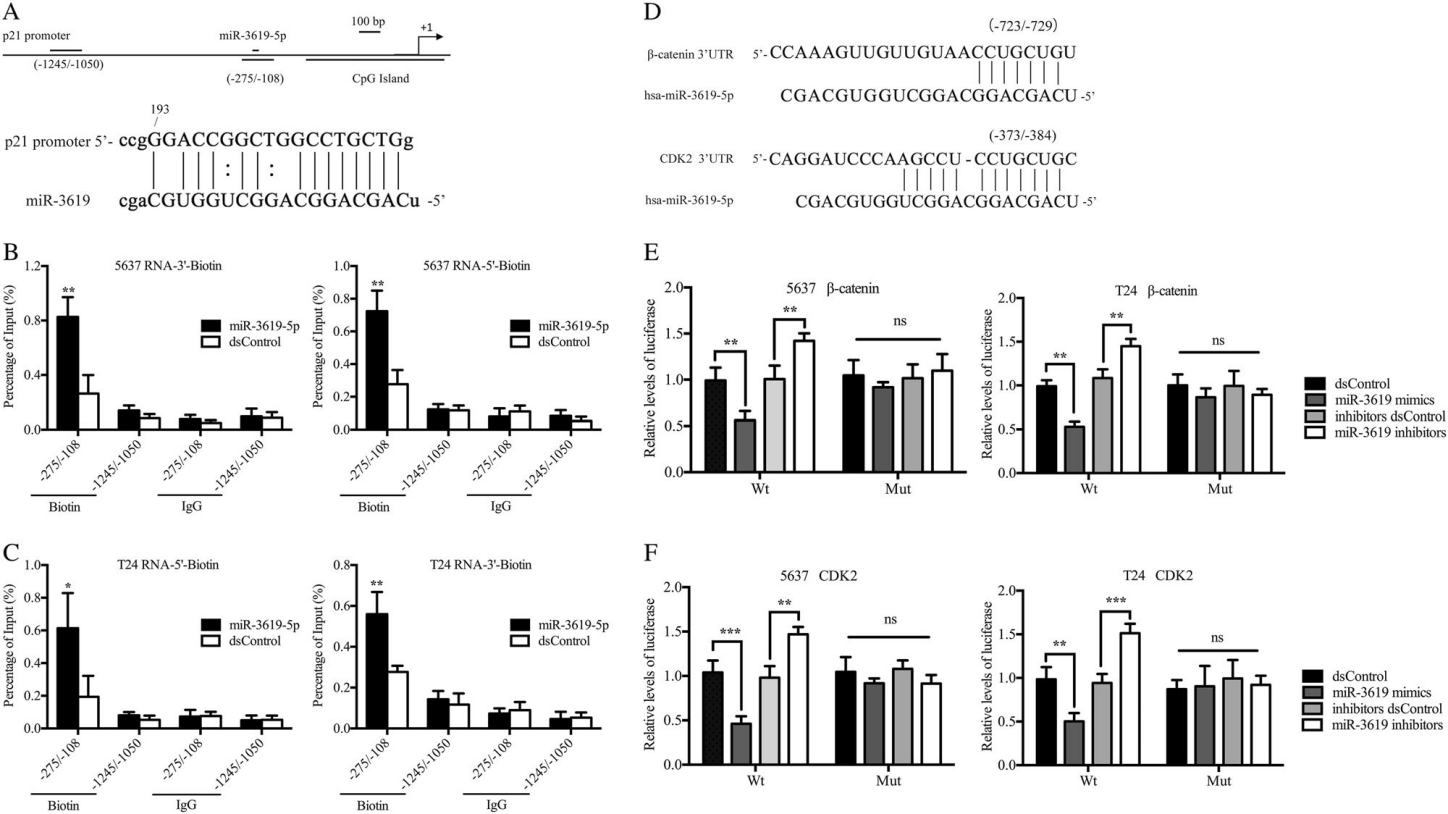
MiR-3619 interacts with the p21 promoter and directly targets β-catenin and CDK2. The specific cells were transfected with 50 nM biotinylated miRNA or dsControl for 72 h. An anti-biotin antibody was used to pull down miRNA-associated DNA. The resulting DNA was amplified by real-time PCR with corresponding primer sets and normalized to input levels. IgG was used as a negative antibody. **a** Schematic illustration of the primers capable of amplifying the p21 promoter at different regions. Locations are shown relative to the TSS. **b**, **c** ChIP assay showed that biotin-labeled miR-3619 pulled down promoter proximal DNA (−275/−108) more effectively than the dsControl RNA. In contrast, there were no differences in the binding of miR-3619 and dsControl RNAs to DNA upstream of the p21 promoter that served as a negative control in 5637 and T24 cells. **d** Predicted miR-3619 target sites in the β-catenin and CDK2 3′-UTRs. Lines indicate perfect matches. **e**, **f** The relative luciferase activity was detected by dual-luciferase reporter assay in the indicated cells. Firefly luciferase values were normalized to Renilla luciferase activity. *P < 0.05, **P < 0.01, and ***P < 0.001 compared to the corresponding dsControl group

Studies have verified that the binding of miRNAs to target gene promoters is a possible mechanism for RNA activation^21–23^. ChIP assay was conducted to identify whether miR-3619 activated p21 by targeting a specific site in its promoter. Seventy-two hours after transfection with biotinylated miR-3619 or the dsControl, the target promoter DNA was pulled down using a corresponding biotin antibody and amplified by qRT-PCR (Fig. 5a). The primer set amplifying the p21 promoter from −1245 bp to −1050 bp relative to the TSS (transcription start site) served as a negative control. As seen in Fig. 5b, c, both the 5′-end and 3′-end of the biotin-labeled miR-3619 pulled down promoter proximal DNA (from −275 to −108) more obviously than the dsControl in 5637 and T24 cells. In contrast, there was no remarkable change in the covalent link of the miR-3619 and the dsControl RNAs to the negative control region (−1245 to −1050). These results indicated that miR-3619 activated p21 gene expression by directly combining with the specific promoter sequence.

Bioinformatics predictions showed that the 3′-UTRs for the human β-catenin and CDK2 mRNAs contain a potential miR-3619 binding site (Fig. 5d). However, direct evidence for the relationship between miR-3619 and β-catenin or CDK2 mRNA by luciferase assay was still required. As shown in Fig. 5e, f, the relative reporter gene luciferase activity was significantly weakened when 5637 and T24 cells were cotransfected with luciferase reporter plasmids containing the predicted β-catenin or CDK2 mRNA target regions and miR-3619 mimics, while the suppression disappeared when the 3′-UTR binding sites were mutated. Moreover, the cotransfection of miR-3619 inhibitors with the wild β-catenin or CDK2 3′-UTR plasmid led to a relative increase in luciferase signal. These data confirmed that β-catenin and CDK2 were direct targets of miR-3619.

### β-catenin and CDK2 knockdown mimic the miR-3619-induced phenotype

There may be a link between the phenotypic changes induced by miR-3619 in BCa cells and the suppression of β-catenin and CDK2. We introduced short interfering RNAs (siRNAs) targeting β-catenin or CDK2 into BCa cell lines. β-catenin or CDK2 knockdown alone or in combination suppressed 5637 and T24 cell growth, reduced the number of colonies and inhibited 5637 and T24 cell proliferation (Supplementary Figures 3A-F). Additionally, β-catenin and/or CDK2 knockout increased both early and late stage apoptosis in 5637 and T24 cells (Supplementary Figure 3G). These results are similar to those of miR-3619 overexpression in 5637 and T24 cells.

We subsequently detected the migration and invasion capacities of BCa cells after transfection with si-β-catenin and/or si-CDK2. As shown in Supplementary Figure 4A-C, transfection of β-catenin and/or CDK2 siRNAs led to retarded wound closing and obviously weakened their migration and invasion abilities compared with the dsControl group cells. Besides, β-catenin knockdown caused a significant decrease in C-myc (Supplementary Figure 4D); CDK2 silencing downregulated MMP9 expression in 5637 and T24 cells. Most importantly, p21 and E-cadherin were upregulated after β-catenin or CDK2 knockdown in the two cell lines (Supplementary Figure 4E). Together, these data suggested that β-catenin and CDK2 inhibition was involved in miR-3619-mediated growth arrest in BCa cells. β-catenin and CDK2 suppression also had a role in miR-3619-triggered migration and invasion in 5637 and T24 cells.

### MiR-3619-induced growth arrest and metastasis inhibition are p21-dependent in BCa cells

**Fig. 6:**
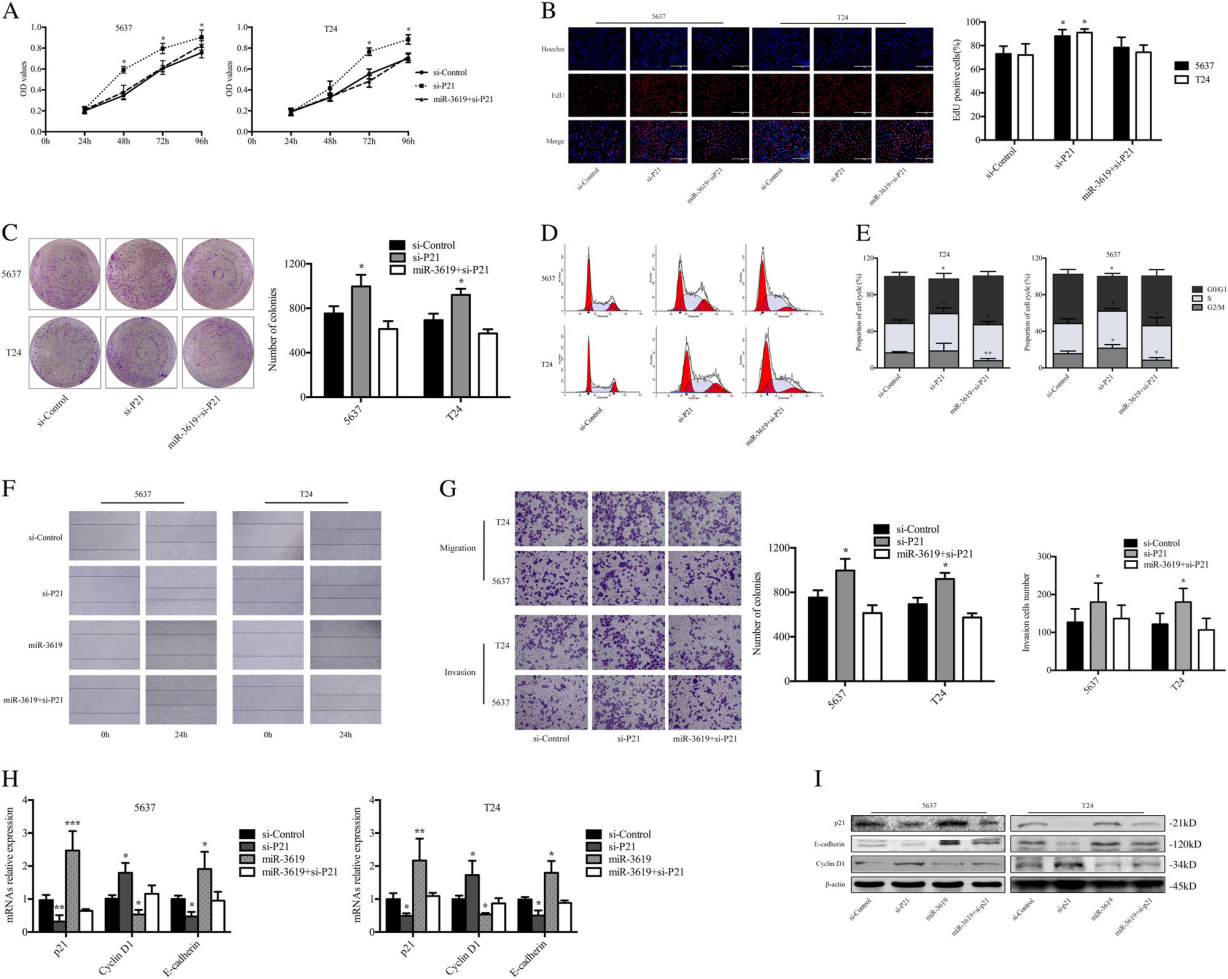
MiR-3619-induced growth arrest and metastasis inhibition are p21-dependent in 5637 and T24 cells. The indicated cells were transfected with siRNAs against p21 or control siRNAs using Lipofectamine RNAiMax and transfected with miRNA mimics after 24 h. P21 knockdown efficiency was evaluated at day 3 after transfection. **a** Cell viability was measured by CellTiter 96® AQueous One Solution Cell Proliferation Assay at 24, 48, 72, and 96 h after miRNA mimic transfection. **b** EdU assay was conducted to detect cell proliferation 3 days after transfection in 5637 and T24 cells. **c** Colony formation assay was conducted to evaluate cell proliferation. Colonies were counted using the ImageJ software. **d**, **e** The cell cycle was examined with FACS analysis and the cell cycle distribution was quantified. **f** Cell migration was investigated with a wound-healing assay and the images were taken at 0 and 24 h. **g** The effect of silencing p21 as well as miR-3619 cotransfected with siP21 on cell invasion and migration was measured by transwell invasion/migration assay (200×). Representative images are shown (left panel). The results represent the mean ± SD in triplicate using the bar graph (right panel). **h**, **i** mRNA and protein expression of p21, Cyclin D1, and E-cadherin were shown in real-time PCR and western blots, respectively. *P < 0.05, **P < 0.01, and ***P < 0.001 compared with the si-Control group

To determine whether there was a relationship between miR-3619-induced inhibition of cell proliferation, migration, and invasion and the expression of p21 in BCa cells, we transfected 5637 and T24 cells with siRNA targeting p21 and miR-3619 mimics, then detected cell proliferation and metastasis. CellTiter 96® AQueous One Solution Cell Proliferation assay and EdU assay was conducted and the outcomes showed that overexpression of miR-3619 failed to inhibit the cell proliferation in p21 knockdown 5637 and T24 cells (Fig. 6a, b). Furthermore, we found colony formation ability of the BCa cells was also restored after cotreatment of si-P21 (Fig. 6c). As shown in Fig. 6d, expression of miR-3619 failed to induce G1/G0 arrest in p21 knockdown 5637 and T24 cells. However, the cells in S phase were increased and the G2/M phase population was decreased in miR-3619 + si-P21 group compared with si-Control group in BCa cells (Fig. 6e). Wound healing assay was conducted and found miR-3619 also failed to inhibit closing the wound within 24 h after knockdown p21expression (Fig. 6f). Furthermore, the transwell assay was performed to further assess cells migration and invasion capacity after knockout p21 genes. As shown in Fig. 6g, depletion of p21 dramatically restored cells migration and invasion in response to miR-3619 transfection. Consistently, as shown in Fig. 6h, i miR-3619 failed to downregulate Cyclin D1 or upregulate E-cadherin after si-p21 cotransfection in 5637 and T24 cells.

### Downregulation of endogenic miR-3619 promotes the growth and metastasis in BCa cells

We knocked down endogenous miR-3619 in BCa cells to further validate miR-3619 regulation of p21, β-catenin, and CDK2. As shown in Supplementary Figure 5A, p21and E-cadherin mRNAs were downregulated and Cyclin D1 mRNA expression was increased after transfection of miR-3619 inhibitor. Moreover, the p21 protein of anti-miR-3619 group was decreased while β-catenin and CDK2 were increased compared with anti-Control group (Supplementary Figure 5B). The immunofluorescence demonstrated that the β-catenin in nucleus was increased after transfection of miR-3619 inhibitor (Supplementary Figure 5C). Besides, growth upregulation was observed upon anti-miR-3619 transfection in cell proliferation assay compared with anti-Control group (Supplementary Figure 5D). Moreover, anti-miR-3619 transfection of 5637 and T24 cells produced a trend towards increased colony formation (Supplementary Figure 5E and 5F). Furthermore, inhibiting miR-3619 led to decrease in cell apoptosis (Supplementary Figure 5G). The result of transwell assay displaying downregulation of miR-3619 remarkably promoted BCa cells capacity of migration and invasion (Supplementary Figure 5H).

## Discussion

Bladder cancer is currently one of the fastest growing tumor in the world. In the past decade, research on metastasis and invasion has been greatly accelerated, which has led to hallmark-targeting therapies development^24^. However, a targeted therapeutic agent may incompletely shut off one or some symbolic abilities by suppressing one crucial pathway in a tumor, allowing partial cancer cells to survive with residual functions until they or their progeny finally accommodate to the selective pressure imposed by the therapy^24,25^. Thus, identifying more important regulators or biomarkers associated with cancer cell proliferation and metastasis and targeting all or most of these supporting pathways therapeutically has become increasingly essential. A few biomarkers have been found to monitor BCa including p21 gene^26^. In this study, we found that p21 gene also plays an important role in miR-3619 inhibition of BCa. Silencing of endogenous p21 gene in tumor cells significantly reduced the effect of miR-3619 in inhibiting bladder tumors.

MiRNAs are known global regulators of gene expression, and dysregulation of miRNA expression and its associated signaling networks has been implicated in tumor progression^27–30^. In this study, we provided evidence that miR-3619 was a candidate BCa inhibitor and was associated with directing the BCa cell phenotype. BCa cell proliferation in vitro and in vivo and metastasis in vitro were dramatically inhibited by miR-3619 through interactions with p21 promoter-specific sequences. We presented data showing that β-catenin and CDK2 were direct targets of miR-3619. Wnt/β-catenin pathway plays a key role in various biological processes, including cell growth, metastasis, and stem cell self-renewal^20,31^. Dysregulation of the Wnt/β-catenin pathway is related to the development of a variety of tumors^32^. We identified that miR-3619 prevented the accumulation of β-catenin in the cytoplasm or its translocation into the nucleus. Moreover, the Wnt target gene c-Myc was also inhibited after miR-3619 overexpression in 5637 and T24 cells, which indicated that miR-3619 inhibited Wnt/β-catenin pathway in BCa cells. EMT process is a crucial initiator and contributor to tumor metastasis. We identified that miR-3619 overexpression enhanced the expression of the epithelial biomarker E-cadherin and suppressed the expression of the mesenchymal biomarkers Vimentin, Snail, and N-cadherin in BCa cells which indicated that miR-3619 suppressed the EMT progression in BCa cells.

The phenomenon of RNA activation (RNAa) was discovered in recent years. Our previous studies have identified that several miRNAs (miR-1236, miR-370, and miR-1180) could activate the expression of the tumor suppressor gene p21^16,33,34^. We found here that endogenous miR-3619 was downregulated in BCa tissues and cells compared with corresponding normal controls. Moreover, miR-3619 overexpression readily upregulated p21 gene expression by targeting a putative site in its promoter. Experimentally enforced expression of miR-3619 in BCa cells resulted in cell cycle arrest, senescence acceleration, increased apoptosis, and metastasis suppression. The downregulation of apoptosis is a key factor in tumorigenesis, cancer progression, and chemotherapy resistance in most cancers types^35^. Thus, miR-3619 fulfilled the criteria of a tumor suppressor gene in the context of BCa.

We provided evidence for p21 involvement in miR-3619-driven cell cycle arrest, senescence, apoptosis, migration, and invasion. Furthermore, miR-3619 also caused a marked change in the p21 downstream genes, Cyclin D1 and E-cadherin in both T24 and 5637 cells. Furthermore, we found that β-catenin and CDK2 met the miR-3619 target criteria to create this phenotype. First, they were both downregulated upon miR-3619 transfection into BCa cells. Second, predicted miR-3619 binding sites in their 3′-UTRs reduced the expression of a reporter construct in the presence of miR-3619.

β-catenin and CDK2 may be linked to negative regulation of the p21 pathway activity. In this study, we found a prominent phenotypic similarity between enforced miR-3619 expression and β-catenin/CDK2 knockdown in two BCa cell lines. We also identified that ectopic expression of β-catenin/CDK2 statistically increased cell proliferation while inhibiting apoptosis in BCa cells. This study demonstrated that β-catenin induced tumorigenicity in BCa cells by activating the Wnt/β-catenin pathway. Furthermore, CDK2 itself also played a crucial role in regulating the proliferation and metastasis of BCa cells. Additionally, the data indicated that β-catenin and CDK2 were upregulated in BCa tissue and several BCa cell lines compared with the dsControl group. Thus, we confirmed that β-catenin and CDK2 as direct target genes of miR-3619, β-catenin and CDK2 played vital roles in BCa cell growth and metastasis. In our experiments, β-catenin and CDK2 expression were significantly downregulated after miR-3619 overexpression in T24 and 5637 cells. Consistently, endogenous miR-3619 knockdown elevated β-catenin and CDK2 levels and reduced p21 levels, as well as changed their downstream targets expression, respectively. Furthermore, p21 knockdown reverted to the changes in the phenotype transcriptome and cellular proteins induced by miR-3619. Together, these data indicated that β-catenin and CDK2 inhibition promoted miR-3619-mediated growth inhibition in 5637 and T24 cells, at least in part through the induction of p21.

In summary, our study found that miR-3619 was decreased in BCa tissues and cell lines and that miR-3619 overexpression inhibited cell growth, invasion, and metastasis. Functional experiments also demonstrated that miR-3619 knockdown promoted BCa cell growth and metastasis. Furthermore, p21, β-catenin, and CDK2 were identified to be direct downstream targets of miR-3619. Taken together, these findings will help us to better understand the mechanisms of miR-3619 in regulating BCa, as well as its role as a potential therapeutic target for the clinical treatment of BCa.

## Materials and methods

### Clinical specimens

The fresh tumor specimens and corresponding para-tumor tissues were collected from 33 patients at Tongji Hospital, Tongji Medical College, Huazhong University of Science and Technology (Wuhan, China) between 2014 and 2016 after informed consent and Ethics Committee’s approval. All the diagnoses were based on pathology reports.

### Cell culture and transfection

The human BCa cell lines J82, 5637, EJ, and T24 (ATCC, USA) were maintained in RPMI 1640 medium (HyClone, USA) with 10% fetal bovine serum (Gibco, USA). Both miRNAs and siRNAs were transfected at a final concentration of 50 nM using Lipofectamine RNAiMax (Invitrogen, USA). Lenti-miR-3619 was used to overexpress miR-3619 following the infection of T24 cells. Lenti-dsControl served as a negative control.

### MiRNA and recombinant lentivirus

The miR-3619-5p mimic, 5′- or 3′- biotin covalently  linked miR-3619 mimic, miR-3619-5p inhibitor, miR-3619-5p mutation as well as interfering RNAs (si-p21, si-β-catenin, si-CDK2 and si-Control) were chemically synthesized by RiboBio Co., Ltd. (Guangzhou, China). Lenti-dsControl and Lenti-miR-3619 were synthesized by GenePharma (Shanghai, China). The sequences of the dsRNA and miRNA were listed in Supplementary Table 1.

### RNA isolation, quantitative real-time PCR, ordinary PCR, and miRNA analysis

Total RNA was extracted with the TRIzol reagent (Invitrogen, USA). Five hundred nanograms of RNA were reverse transcribed into cDNA. The quantitative real-time PCR was performed on an Mx3000P instrument (Stratagene, USA) with SYBR Premix Ex Taq II (Takara, China). The primers were provided by Invitrogen (Shanghai, China) and were listed in Supplementary Table 2. The partial mRNA PCR product was also analyzed on 2% agarose gels and visualized.

### Western blotting analysis

Total tissues and cellular proteins were extracted and separated by 10% sodium dodecyl sulfate polyacrylamide gel electrophoresis (SDS-PAGE) and then transferred onto polyvinylidene fluoride membranes (Millipore, USA). The membranes incubated overnight at 4 °C with primary antibodies. The antibodies against CDK2, Cyclin D1, β-catenin, C-myc, N-cadherin, Snail were purchased from Affinity (USA), p21 antibody was from Cell Signaling Technology (USA), E-cadherin antibody was from BD Biosciences (USA), antibodies against MMP9, Vimentin, β-actin and GAPDH were from Boster (China).

### Immunohistochemistry (IHC) analysis

The tissues were fixed in 4% paraformaldehyde, routinely dehydrated, paraffin-embedded tissue sections (5 μm) were deparaffinized and rehydrated. Paraffin sections were incubated with primary antibodies against β-catenin (Affinity, USA), CDK2 (Affinity, USA), and p21 (Cell Signaling Technology, USA). The sections were stained with hematoxylin as counterstaining.

### Chromatin immunoprecipitation assay (ChIP assay)

Cells transfected with biotin-labeled miRNAs were used for ChIP assay using a ChIP assay kit (Millipore) at 72 h. Collection of 3 × 10^6^ cells were used for a single immunoprecipitation. One percent formaldehyde was used to cross-link chromatin for 10 min at 37 °C. Then, SDS lysis buffer was used to wash and re-suspend the fixed cells. Biotin (Santa Cruz Biotechnology, USA) antibody and normal rabbit IgG (Millipore, USA) (negative control) were used for immunoprecipitation after the chromatin was precleared with protein A agarose/Salmon sperm DNA overnight at 4 °C. Then, the antibody/antigen/DNA complexes were collected and reversed. The column-purified (Omega Bio-tek, USA) DNA was used as a template for real-time PCR. The sequences of primers were listed in Supplementary Table 2.

### Immunofluorescence staining

Transfected BCa cells were fixed with 4% paraformaldehyde, 0.2% Triton X-100 was used to permeabilize cells, 5% BSA was used to block the cells. The BCa cells were incubated with primary antibody overnight at 4 °C. Then, the cells were incubated with the secondary antibodies for 1 h at 37 °C. The coverslips were stained with 4′,6-diamidino-2-pheny-lindole (DAPI) after washing three times to visualize the nucleus.

### Cell proliferation assay

CellTiter 96® AQueous One Solution Cell Proliferation Assay kit (Promega, USA) was used to assess cell growth as previously described^36^.

### Clonogenic survival assay

The colonies were fixed 10 days later and stained with 0.5% crystal violet (Sigma, USA) for 30 min at room temperature. The following formula was used to determine the colony formation rate: Colony formation rate = number of colonies / number of seeded cells × 100%.

### 5-Ethynyl-2′-deoxyuridine proliferation assay

The Cell-Light EdU DNA cell kit (Ribobio, China) was used to detect cell proliferation. The cells were incubated with 50 mM 5-ethynyl-2′-deoxyuridine (EdU) for 3 h at 37 °C. The fixed cells were treated with 0.5% Triton X-100 for 15 min; 1 mg/ml DAPI (Sigma, USA) was used to stain the cell nuclei for 30 min. The EdU-labeled cells were observed by fluorescence microscopy.

### Cell cycle and apoptosis analysis by flow cytometry

The fixed cells by 70% cold ethanol were treated with RNase A (Sigma, USA) at 37 °C for 30 min and stained with a propidium iodide (PI) (Key-gen Biotech, China) staining solution at 4 °C for 30 min. Next, the cells were analyzed on an FACS flow cytometer (BD Biosciences, USA). After co-staining the cells with Annexin V-FITC and PI, the cell apoptosis was assessed by flow cytometry according to the manufacturer’s instructions (BD Biosciences, USA).

### Wound-healing assay

About 5 × 10^5^ BCa cells were plated in a six-well plate following transfection. The confluent cell monolayers were scratched. Then, PBS was used to wash the nonadherent cells and serum-free medium was added to the wells. The migrated distances were observed at 0 and 24 h post-scratch.

### Migration and invasion assay

A 24-well Boyden chamber with an 8 μm pore size polycarbonate membrane (Corning, USA) was used to detect the cell motility. For the invasion assay, Matrigel (BD Biosciences, USA) was precoated into the membranes to form a matrix barrier. The membranes were fixed and stained with 0.5% crystal violet (Sigma, USA).

### Subcutaneous xenograft models

Approximate 5 × 10^6^ infected T24 cells were subcutaneously injected into the right back of 4-week-old male BALB/c-nude mice (Hua Fukang Biological Technology Co., Ltd, China). A caliper was used to measure the tumor length and width every 4 days for 30 days. The xenograft tumor volume was calculated with the following formula: *V* = (length × width^2^)/2. On day 30, the tumors were removed and weighed. All the experimental nude mice were cared for and manipulated according to the NIH Animal Care and Use Committee Guidelines of the Experiment Animal Center of Tongji Medical College of Huazhong University of Science and Technology (Wuhan, China).

### Statistical analysis

Data are presented as the mean ± standard deviation (SD) for three independent experiments. Differences between groups were analyzed by the two-tailed Student’s *t* test using SPSS version 22.0 software (SPSS Inc., Chicago, IL, USA). Statistical significance among three or more groups was based on one-way ANOVA. The correlation between variables was analyzed using Spearman’s correlation test. Survival curves were constructed by the Kaplan−Meier method to simultaneously adjust all potential prognostic variables. A *P* value < 0.05 was considered to be statistically significant.

## Electronic supplementary material

Supplementary table 1

Supplementary table 2

Supplementary Figure 1

Supplementary Figure 2

Supplementary Figure 3

Supplementary Figure 4

Supplementary Figure 5

Supplementary Figure Legends
