# Supplementary material for: MicroRNA-3619-5p suppresses bladder carcinoma progression by directly targeting β-catenin and CDK2 and activating p21
Source: Cell Death Dis. 2018 Sep 20;9(10):960. doi: 10.1038/s41419-018-0986-y (PMC6147790; doi:10.1038/s41419-018-0986-y)
Supplement: Supplementary file 1 — Supplementary table 1 [file 41419_2018_986_MOESM1_ESM.doc]

Supplementary table 1. Sequences for dsRNA and siRNA used in present study

| Synthesized RNAs | RNA sequences |
| --- | --- |
| miR-3619 (S) | GCUGCACCAGCCUGCCUGCUGA |
| miR-3619 (AS) | UCAGCAGGCAGGCUGGUGCAGC |
| dsControl (S) | ACUACUGAGUGACAGUAGA[dT][dT] |
| dsControl (AS) | UCUACUGUCACUCAGUAGU[dT][dT] |
| si-p21 (S) | CUUCGACUUUGUCACCGAG |
| si-p21 (AS) | CUCGGUGACAAAGUCGAAG |
| si-β-catenin(S) | CAGGGGGUUGUGGUUAAGCUCUU |
| si-β-catenin (AS) | AAGAGCUUAACCACAACCCCCUG |
| si-CDK2 (S) | UCCUUAAGGAGCUCAA[dT][dT] |
| si-CDK2 (AS) | UUGAGCUCCUUAAGGA[dT][dT] |
| si-Control (S) | UUCUCCGAACGUGUCACGU[dT][dT] |
| si-Control (AS) | ACGUGACACGUUCGGAGAA[dT][dT] |
