# Supplementary material for: MicroRNA-3619-5p suppresses bladder carcinoma progression by directly targeting β-catenin and CDK2 and activating p21
Source: Cell Death Dis. 2018 Sep 20;9(10):960. doi: 10.1038/s41419-018-0986-y (PMC6147790; doi:10.1038/s41419-018-0986-y)
Supplement: Supplementary file 2 — Supplementary table 2 [file 41419_2018_986_MOESM2_ESM.doc]

Supplementary table 2. Primers used in this study

| Name | Sequences (5’-3’) | Assay used for |
| --- | --- | --- |
| p21(S) | GCCCAGTGGACAGCGAGCAG | PCR |
| p21(AS) | GCCGGCGTTTGGAGTGGTAGA | PCR |
| E-cadherin (S) | ACCAGAATAAAGACCAAGTGACCA | PCR |
| E-cadherin (AS) | AGCAAGAGCAGCAGAATCAGAAT | PCR |
| GAPDH (S) | TCCCATCACCATCTTCCA | PCR |
| GAPDH (AS) | CATCACGCCACAGTTTCC | PCR |
| Cyclin D1 (S) | GCTGCGAAGTGGAAACCATC | PCR |
| Cyclin D1 (AS) | CCTCCTTCTGCACACATTTGAA | PCR |
| N-cadherin (S) | AGC- CAACCTTAACTGAGGAGT | PCR |
| N-cadherin (AS) | GGCAAGTTGATTGGAGGGATG | PCR |
| Vimentin (S) | GACGCCATCAACACCGAGTT | PCR |
| Vimentin (AS) | CTTTGTCGTTGGTTAGCTGGT | PCR |
| Snail (S) | ACCCCACATCCTTCTCACTG | PCR |
| Snail (AS) | TACAAAAACCCACGCAGA CA | PCR |
| p21-275/-108 (S) | GGAAATGTGTCCAGCGCACC | CHIP |
| p21-275/-108 (AS)  p21-1245/-1050 (S)  p21-1245/-1050 (AS) | AGCGCGGCCCTGATATACAACC  GCTGCGAAGTGGAAACCATC  CCTCCTTCTGCACACATTTGAA | CHIP  CHIP  CHIP |
| GAPDH (S) | TACTAGCGGTTTTACGGGCGCACGT | CHIP |
| GAPDH (AS) | TCGAACAGGAGGAGCAGAGAGCGAA | CHIP |
| U6(F) | CTCGCTTCGGCAGCACATA | PCR |
| U6(R) | CGAATTTGCGTGTCATCCT | PCR |
