# Supplementary figures and images for: MicroRNA-3619-5p suppresses bladder carcinoma progression by directly targeting β-catenin and CDK2 and activating p21
Source: Cell Death Dis. 2018 Sep 20;9(10):960. doi: 10.1038/s41419-018-0986-y (PMC6147790; doi:10.1038/s41419-018-0986-y)

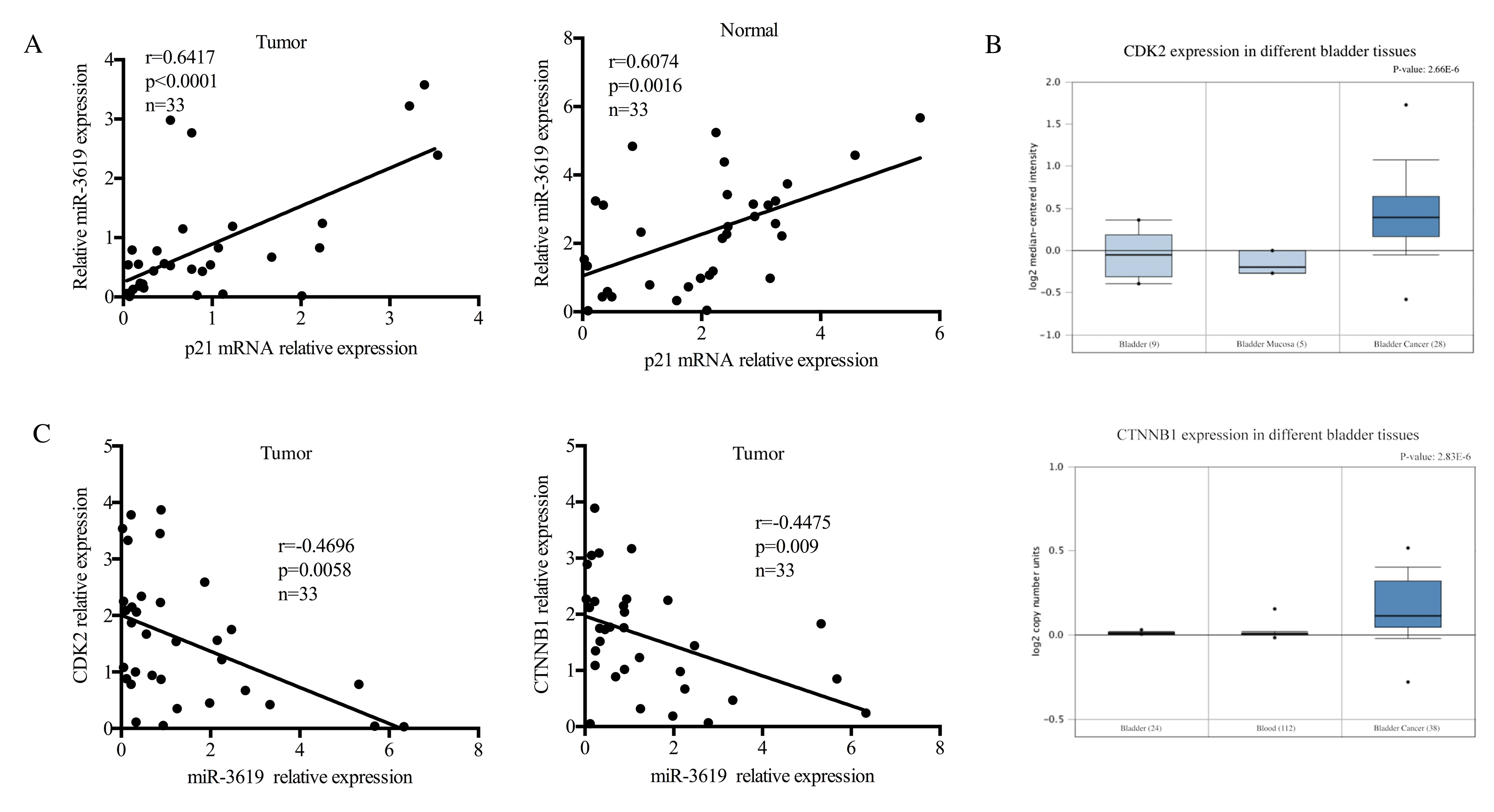

Supplement: Supplementary file 3 — Supplementary Figure 1 [file 41419_2018_986_MOESM3_ESM.tif]

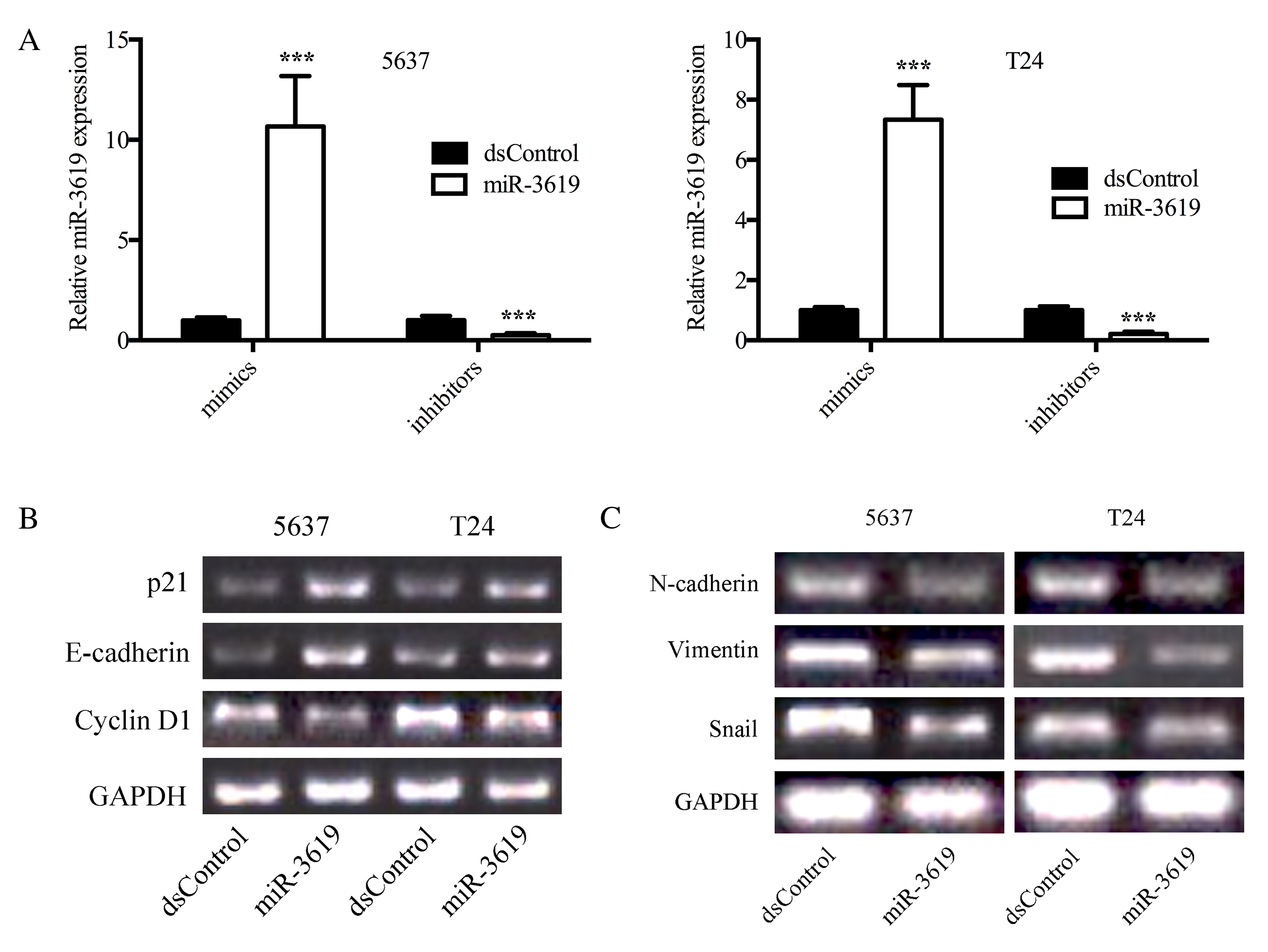

Supplement: Supplementary file 4 — Supplementary Figure 2 [file 41419_2018_986_MOESM4_ESM.tif]

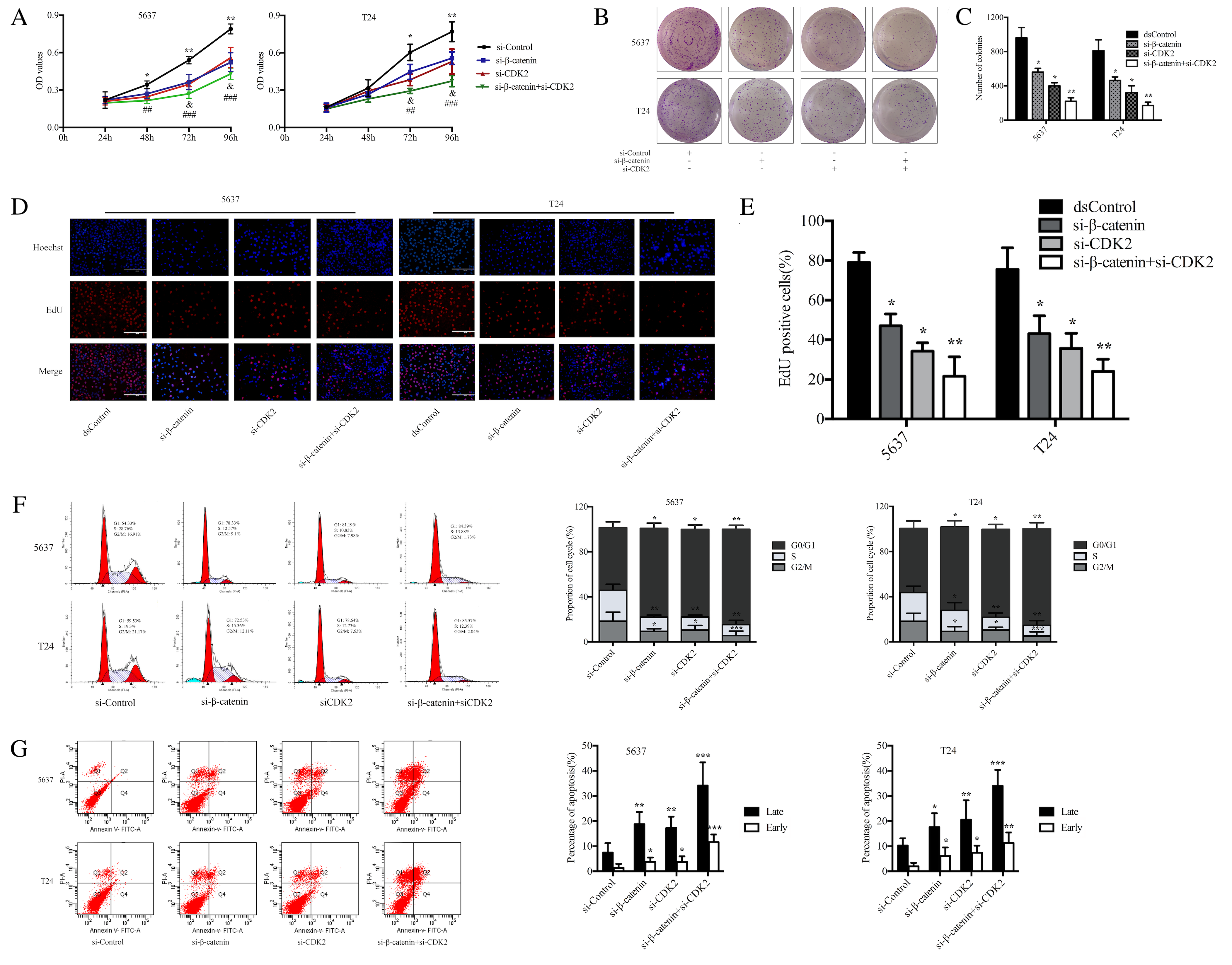

Supplement: Supplementary file 5 — Supplementary Figure 3 [file 41419_2018_986_MOESM5_ESM.tif]

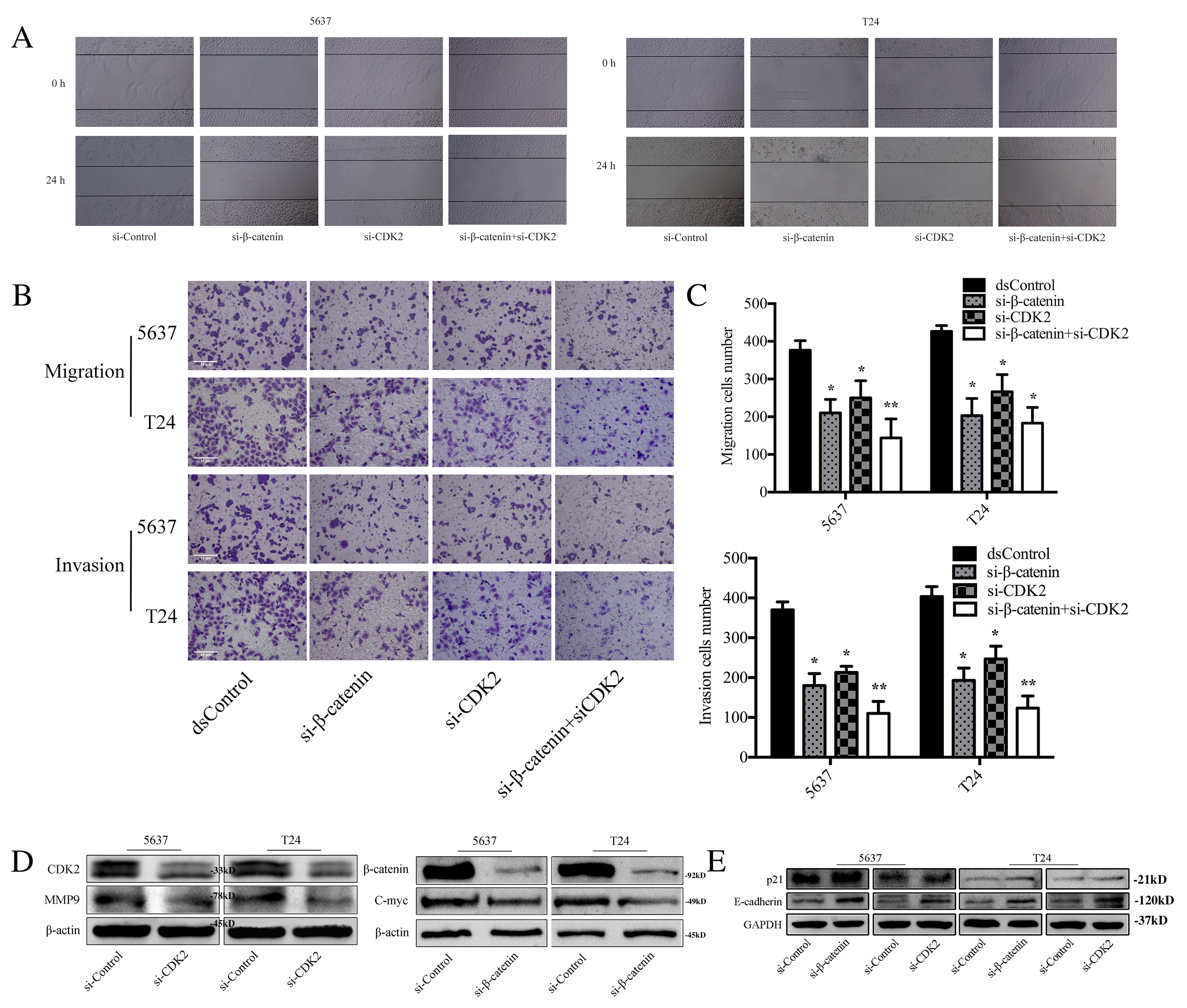

Supplement: Supplementary file 6 — Supplementary Figure 4 [file 41419_2018_986_MOESM6_ESM.tif]

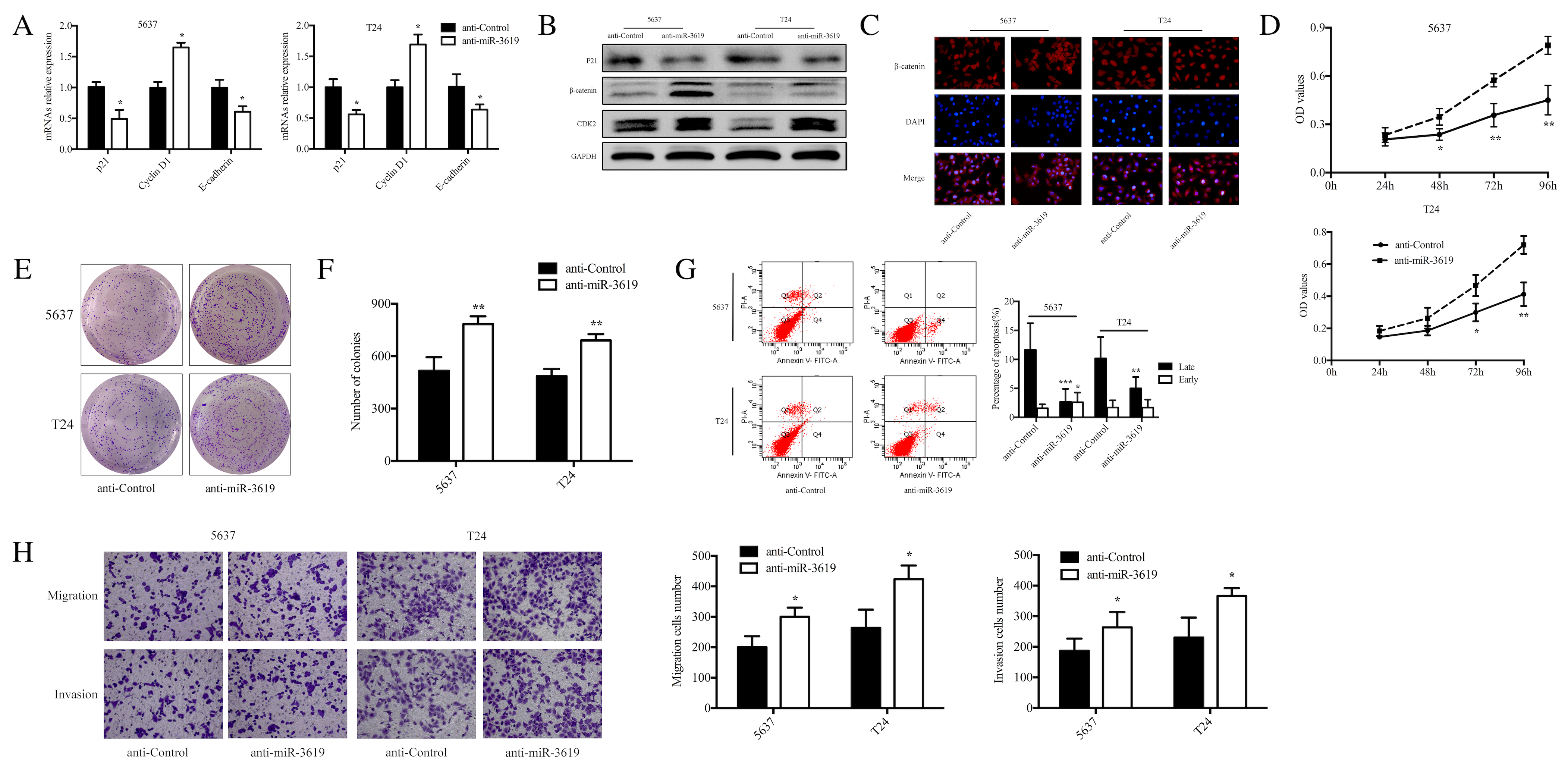

Supplement: Supplementary file 7 — Supplementary Figure 5 [file 41419_2018_986_MOESM7_ESM.tif]
