# Supplementary material for: MicroRNA-3619-5p suppresses bladder carcinoma progression by directly targeting β-catenin and CDK2 and activating p21
Source: Cell Death Dis. 2018 Sep 20;9(10):960. doi: 10.1038/s41419-018-0986-y (PMC6147790; doi:10.1038/s41419-018-0986-y)
Supplement: Supplementary file 8 — Supplementary Figure Legends [file 41419_2018_986_MOESM8_ESM.doc]

**MicroRNA-3619-5p suppresses bladder carcinoma progression by directly**

**targeting β-catenin and CDK2 and activating p21**

Qingsong Zhang^1^, Shuo Miao^2^, Xihong Han^3^, Chuanchang Li^1^, Mengyang Zhang^1^, Kai Cui^1^, Tao Xiong^1^, Zhong Chen^1, *^, Chenghe Wang^4^, Hua Xu^1^

1: Department of Urology, Tongji Hospital, Tongji Medical College, Huazhong University of Science and Technology, No. 1095 JieFang Avenue, Wuhan 430030, Hubei, China

2: Department of Pharmacology, Tongji Medical College, Huazhong University of Science and Technology, No. 1095 JieFang Avenue, Wuhan 430030, Hubei, China

3: Department of Cardiology, Shouguang People's Hospital, Shouguang, 262700, Shandong, China.

4: Department of Urology, Ruijin Hospital, School of Medicine, Shanghai Jiaotong

University, Shanghai 200025

*: Corresponding Author: Zhong Chen, MD, PhD, Department of Urology, Tongji Hospital, Tongji Medical College, Huazhong University of Science and Technology, No. 1095 Jie Fang Avenue, Wuhan 430030, Hubei, China. Tel: (86) 027-83663673; Fax: (86) 027-83663673; E-mail: chenzhongtongji@126.com.

**Supplementary Figure 1** The correlation analysis of miR-3619 and p21 expression levels in BCa and adjacent normal tissues as well as miR-3619 and CDK2 and CTNNB1 expression in BCa tissues. (A) Statistical analysis was performed using the Pearson correlation coefficient analysis, with r and P values as indicated. Positive correlation between miR-1236 and p21 expression levels in BCa and adjacent normal tissues. (B) The expression levels of CDK2 and CTNNB1 analyses using Oncomine (www.oncomine.org) from original published data. CDK2 and CTNNB1 were significantly overexpressed in BCa samples in comparison with normal samples. (C) A correlation test in the resected BCa specimens from 33 patients showing a strong inverse correlation between miR-3619 and CDK2 and CTNNB1.

**Supplementary Figure 2** Transfection ratio was tested by qRT-PCR, the mRNA expression of p21 and its downstream genes E-cadherin and Cyclin D1 as well as EMT related genes were detected by reverse transcription PCR (RT-PCR) in miR-3619 transfected cells. (A) qRT-PCR analysis of the relative miR-3619 levels in 5637 and cells transfected with mimics or inhibitors. (B and C) The expression of p21 and its downstream genes E-cadherin and Cyclin D1 mRNAs as well as EMT related genes N-cadherin, Vimentin and Snail were detected by RT-PCR in miR-3619 transfected cells. GAPDH served as a loading control. *** P<0.001 compared with dsControl group.

**Supplementary Figure 3** β-catenin and CDK2 knockdown mimic miR-3619-induced cells proliferation inhibition. T24 and 5637 cells were transfected with anti-β-catenin (50 nM), anti-CDK2 (50 nM), anti-β-catenin (25 nM) + anti-CDK2 (25 nM) siRNAs or control siRNA (50 nM) with Lipofectamine RNAiMax. (A) 5637 and T24 cells were measured from day 1 to 4 following transfection using the CellTiter 96® AQueous One Solution Cell Proliferation Assay kit. Results were plotted as OD values. Knockdown β-catenin or CDK2 significantly inhibited cells growth compared with si-Control group. *P<0.05, **P<0.01. While silencing β-catenin and CDK2 together, BCa cells growth was remarkably suppressed. ##P<0.01, ###P<0.001 compared with si-Control group. &P<0.05 compared with si-β-catenin or si-CDK2 group. (B) The effect of mimicking overexpression of miR-3619 on cell proliferation was measured by colony formation assay. (C) Quantification of the cell colonies formation. *P<0.05, **P<0.01 compared with dsControl group. (D) EdU assay was conducted to detect cells proliferation 3 days after transfection in 5637 and T24 cells. *P<0.05, **P<0.01 compared with dsControl group. (E) Quantification of EdU-positive cells. (F) Representative images of cell cycle analysis in both 5637 and T24 cells and quantification of cell cycle distribution. (G) Representative flow cytometry images of cell apoptosis. Percentage of early and late apoptotic cells was shown by histogram. *P<0.05, **P<0.01, ***P<0.001 compared with si-Control group.

**Supplementary Figure 4** β-catenin and CDK2 knockdown mimic miR-3619-induced BCa cells migration and invasion suppression. T24 and 5637 cells were transfected with anti-β-catenin (50 nM), anti-CDK2 (50 nM), anti-β-catenin (25 nM) + anti-CDK2 (25 nM) siRNAs or control siRNA (50 nM) with Lipofectamine RNAiMax. (A) The migration ability of the cells was investigated with the wound healing assay and the images were pictured at 0 and 24 h. (B) The migration and invasion ability of BCa cells was identified by using the transwell assay (200×). Quantitative analysis of the total migrated and invasive cells from three independent experiments was shown in (C). *P<0.05, **P<0.01 compared with dsControl group. (D and E) Protein expression of p21, CDK2, β-catenin and their downstream genes from siRNA-transfected cells is shown in western blots.

**Supplementary Figure 5** Down-regulation of miR-3619 promotes the growth and metastasis in BCa cells. We used the inhibitor of miR-3619 to simulate the down-regulation of miR-3619 in T24 and 5637 cells. (A) mRNA expression of p21, Cyclin D1 and E-cadherin in anti-miR-transfected 5637 and T24 cells is shown in real-time PCR. (B) The expression of p21, β-catenin and CDK2 proteins in anti-miR-transfected 5637 and T24 cells is shown in western blot. (C) Immunofluorescence staining of β-catenin in anti-miR-transfected 5637 and T24 cells. (D) Cell viability was measured by CellTiter 96® AQueous One Solution Cell Proliferation Assay for 24, 48, 72 and 96 h after anti-miR-3619 transfection. (E) Colony formation assay was conducted to evaluate the cells proliferation. (F) Colonies were counted using ImageJ software. (G) The effect of anti-miR-3619 on cell apoptosis was detected by flow cytometry analysis. (H) The effect of anti-miR-3619 on cell invasion and migration was measured by transwell invasion/migration assay (200×). Results represented the mean ± SD in triplicate using bar graph. *P<0.05, **P<0.01, ***P<0.001 compared with anti-Control group.
